# Prevalence and Temporal Trends of Mental Disorders in Persons with Opioid Use Disorder and Concurrent Mental Disorders in British Columbia, Canada, Using Population-Level Administrative Data, 2013 to 2021: Prévalence et tendances temporelles des troubles mentaux chez les personnes souffrant d’un trouble lié à la consommation d’opioïdes et de troubles mentaux concomitants en Colombie-Britannique, au Canada, à partir de données administratives au niveau de la population, entre 2013 et 2021

**DOI:** 10.1177/07067437251347150

**Published:** 2025-06-09

**Authors:** Angela Russolillo, Fahmida Homayra, Bohdan Nosyk

**Affiliations:** 1 Centre for Advancing Health Outcomes, Vancouver, BC, Canada; 2 School of Nursing, University of British Columbia, Vancouver, BC, Canada; 3 Providence Health Care, Mental Health Program, Vancouver, BC, Canada; 4 Centre for Cardiovascular Innovation, Department of Medicine, University of British Columbia, Vancouver, BC, Canada; 5 Faculty of Health Sciences, 1763 Simon Fraser University, Burnaby, BC, Canada

**Keywords:** mental disorders, opioid use disorder, anxiety, depression, epidemiology prevalence, population data

## Abstract

**Objective:**

Opioid use is a major public health issue and associated with a broad range of comorbid mental disorders. Globally, there is considerable variability in reported rates of mental disorders among individuals with opioid use disorder (OUD), limiting timely intervention and evidence-based treatment among this population. We estimate the prevalence of specific mental disorders among individuals with a concurrent OUD using population-level administrative data in British Columbia, Canada.

**Method:**

A population-based retrospective observational study using individual-level linked health administrative data in British Columbia, Canada. Individuals with an OUD and concurrent mental disorder between January 1, 2013, and August 31, 2021, were included and followed from their first indication of OUD until censoring (death, administrative loss to follow-up, or August 31, 2021). We reported annual period (2013-2021) prevalence rates and age-standardized prevalence rates per 100,000 population (stratified by sex).

**Results:**

The population included 73,855 individuals (female 40.6%, median age, 36 [27-48]) with an OUD and concurrent mental disorder. During the observation period anxiety disorders were the most prevalent (91.7%) mental disorders followed by depression (73.6%), bipolar disorder (35.3%), schizophrenia spectrum disorders (20.4%), and personality disorders (19.5%). Among the population, the annual period prevalence of any mental disorder increased from 35,603 in 2013 to 60,940 in 2021, with an average annual percent difference of 7.0%, driven by increases in schizophrenia spectrum disorders and attention deficit/hyperactivity disorder. Overall, the annual age-standardized prevalence of any mental disorder was higher among males.

**Conclusions:**

Our findings demonstrate a steadily growing prevalence of people with OUD and a concurrent mental disorder and emphasize the need for access to mental disorder treatment among this population. Estimating specific mental disorder prevalence is a pragmatic step toward informing clinical guidelines, service needs, and health system planning.

## Résumé

**Objectif:**

La consommation d’opioïdes est un problème de santé publique majeur associé à un large éventail de troubles mentaux comorbides. À l’échelle mondiale, les taux déclarés de troubles mentaux chez les personnes souffrant d’un trouble lié à la consommation d’opioïdes varient considérablement, ce qui limite l’intervention en temps opportun et le traitement basé sur des données probantes au sein de cette population. Nous estimons la prévalence de troubles mentaux spécifiques chez les personnes atteintes d’un trouble lié à la consommation d’opioïdes à l’aide de données administratives à l’échelle de la population en Colombie-Britannique, au Canada.

**Méthodes:**

Étude d’observation rétrospective basée sur la population utilisant des données administratives de santé individuelles liées entre elles en Colombie-Britannique, au Canada. Les personnes présentant un trouble lié à la consommation d’opioïdes et un trouble mental concomitant entre le 1er janvier 2013 et le 31 août 2021 ont été incluses et suivies depuis leur première indication de trouble lié à la consommation d’opioïdes jusqu'a la fin d'inscription (décès, perte de suivi administratif jusqu’au suivi ou le 31 août 2021). Nous avons rapporté les taux de prévalence annuels pour la période 2013-2021 et les taux de prévalence normalisés selon l’âge pour 100 000 habitants (stratifiés selon le sexe).

**Résultats:**

La population comprenait 73 855 personnes (40,6% de femmes, âge médian de 36 ans [27-48]) présentant un trouble lié à la consommation d’opioïdes et un trouble mental concomitant. Au cours de la période d’observation, les troubles anxieux étaient les troubles mentaux les plus fréquents (91,7%), suivis de la dépression (73,6%), des troubles bipolaires (35,3%), des troubles du spectre de la schizophrénie (20,4%) et des troubles de la personnalité (19,5%). Au sein de la population, la prévalence annuelle de tout trouble mental est passée de 35 603 en 2013 à 60 940 en 2021, avec une différence annuelle moyenne de 7,0%, due à l’augmentation des troubles du spectre de la schizophrénie et du trouble du déficit de l’attention. Dans l’ensemble, la prévalence annuelle normalisée selon l’âge de tout trouble mental était plus élevée chez les hommes.

**Conclusions:**

Nos résultats démontrent une prévalence de plus en plus élevée de personnes atteintes d’un trouble lié à la consommation d’opioïdes et d’un trouble mental concomitant, et soulignent la nécessité d’avoir accès à un traitement pour les troubles mentaux au sein de cette population. L’estimation de la prévalence de certains troubles mentaux est une mesure pragmatique qui permettra d’éclairer les directives cliniques, les besoins en matière de services et la planification du système de santé.

## Introduction

North America is currently in the midst of an unprecedented opioid epidemic. In Canada, the number of fatal overdoses has increased over the past two decades,^1,2^ with nearly 50,000 opioid-related deaths between 2016 and 2024.^3^ This increase has accelerated in recent years, driven by the growing prevalence of synthetic opioids like fentanyl in the illicit drug market.^4^ Canadian surveillance data estimate that nearly 350,000 people met criteria for opioid use disorder (OUD) and 930,000 reported misusing prescription opioids in 2015.^5^ Many individuals with OUD present with several comorbidities including co-occurring psychiatric and other substance use disorders.^6^ These factors contribute to several cross-cutting health and social challenges, such as homelessness^7^ and incarceration,^8^ often limiting treatment initiation and increasing opioid-related harms.^9^

Opioid use is robustly associated with a broad range of comorbid psychiatric disorders,^10,11^ with evidence of a bidirectional relationship between conditions.^12^ While mental disorders are widely accepted as risk factors for OUD, a recent review suggests that opioid use, over long periods and in higher doses, increases the risks of developing depressive, bipolar, and anxiety disorders.^13^ Moreover, the etiology and pathology of these disorders is complex, characterized by periods of remittance and relapse, and accompanied by significant diagnosis-treatment disparity.^14,15^ In a nationally representative sample of individuals with OUD in the United States, 47% of individuals with mild/moderate mental illness received no mental health treatment and 30% reported unmet mental health needs.^16^

Previous studies of psychiatric comorbidity in patients with OUD have been limited to national survey samples^16,17^ or patients already engaged in OUD treatment in specialty settings.^18–20^ Current estimates have relied on subpopulations such as offenders,^21^ generalized nosology for classification of groups,^22^ or studies spanning short observation periods,^23^ limiting generalizability and potentially underestimating the disorder-specific prevalence and morbidity over time. As public health agencies across North America grapple with how to reduce the burden of the opioid epidemic, the importance of understanding an individual's psychiatric comorbidities in the context of their OUD cannot be overstated.^24^ Failure to describe the breadth and severity of psychiatric illness further limits the ability of public health practitioners, clinicians, and policymakers to appropriately target and resource policy, programmatic, and clinical interventions to meet the needs of this high-risk and clinically complex population.^25^

To address these gaps, we aim to estimate the prevalence of co-occurring psychiatric disorders among individuals with an OUD using population-level administrative data spanning nearly a decade (2013-2021), in British Columbia (BC), Canada.

## Method

### Study Population and Data Sources

We conducted a retrospective observational study using provincial-level linked databases including the Client Roster (demographics),^26^ Medical Services Plan database (physician billings),^27^ the Discharge Abstract Database (hospitalizations),^28^ National Ambulatory Care Reporting System (emergency department visits),^29^ the BC PharmaNet database (drug dispensations),^30^ BC Vital Statistics (deaths),^31^ the Perinatal Services BC database (maternal and infant health outcomes, including substance use during pregnancy),^32^ BC corrections (incarceration in provincial prisons),^33^ the Social Development and Poverty Reduction database (homelessness and income assistance),^34^ and BC Coroners Service database (illicit drug poisoning deaths).^35^ We used a case-finding algorithm to identify the population of individuals aged 18 and older with an OUD and concurrent mental disorder from January 01, 2013 to August 31, 2021. The case-finding algorithm used a combination of data sources, to minimize misclassification due to errors in the coding of physician billing records. Further details on data sources and applications in defining our study population are described in eTables 1, 2, and 3 in the Supplemental Material. Additional information and descriptions of these component datasets are described elsewhere.^36,37^

We applied a case-finding algorithm to identify people with OUD (any opioid agonist treatment [OAT] receipt, hospitalization, emergency department visit, perinatal record, more than two physician billing records, or OUD-related death record between January 1996 and August 2021) and any mental disorder (ever diagnosed between January 1996 and August 2021). We defined exposure to OAT as any receipt of methadone, buprenorphine/naloxone, slow-release oral morphine, or injectable OAT (hydromorphone or diacetylmorphine), and these were differentiated from opioids used for indications of pain control. International Classification of Diseases, Ninth Revision (ICD-9) and International Classification of Diseases, 10th Revision, Canada (ICD-10-CA) codes for each mental disorder type along with hospitalization, emergency department visits, perinatal records, and physician billing records or death records, were used to identify individuals who were ever diagnosed with a mental disorder including anxiety, stress and adjustment disorder, depression, bipolar disorder, personality disorder, schizophrenia spectrum disorders (schizophrenia, schizotypal, and delusional disorders), attention-deficit/hyperactivity disorder (ADHD), and developmental disorders. ICD-10-CA codes for drug-induced psychosis (F19.5, F19.7) were included in the case-finding algorithm for nonopioid substance use comorbidities (see eTable 3 in the Supplemental Material), but not as an indicator of any mental disorder. We utilized validated case-finding algorithms for OUD^37–39^ and for specific mental disorders, including depression, bipolar disorder, and schizophrenia.^40–42^ The algorithm for OUD was subject to sensitivity analyses enhancing case identification and definitions while acknowledging the potential for misclassification.^43^ Further details and definitions for the case-finding algorithm, including diagnostic codes, are described in eTables 1 and 2 in the Supplemental Material.

### Follow-up

Individuals were followed from their first indication of OUD (18th birthday when OUD was identified at age <18, or January 01, 2013 when OUD was identified before the study started) to either the end of follow-up (August 31, 2021), administrative loss to follow-up (no healthcare records for 66 months)^36,44^ or death.

### Measures

We measured demographic characteristics including age, sex, and region of residency (urban/rural); socioeconomic characteristics including history of unstable housing, receipt of income assistance, and history of incarceration; related comorbidities including alcohol use disorder, nonopioid nonalcohol substance use disorders, noncancer chronic pain, hepatitis C, and Human Immunodeficiency Virus/Acquired Immunodeficiency Syndrome (HIV/AIDS) (case-finding algorithm is defined in eTable 3). We measured treatment history including years since OUD diagnosis, engagement in OAT, years since mental disorder diagnosis, psychotropic drug dispensations including antidepressants, antipsychotics, mood stabilizers, antianxiety, and stimulants (medications indicated for each condition are presented in eTable 4). We summarized healthcare utilization (including acute care and non-OAT outpatient care visits for all causes, as well as for drug-related or psychiatric causes) and all-cause mortality.

### Statistical Analysis

We first presented descriptive statistics of demographics, prevalence of specific mental disorders, other comorbidities, and OUD and mental disorder-related healthcare utilization among individuals with an OUD and concurrent mental disorders from 2013 to 2021. Second, we presented the annual period prevalence and age-standardized prevalence rates per 100,000 population by specific mental disorders (overall population and sex-stratified). The period prevalence estimates reflect the proportion of individuals with OUD and concurrent mental disorders (any and specific mental disorders) identified through our case-finding algorithm. We used the 2011 Census of Canadian Population data (age grouping: 18-24; 25-34; 35-44; 45-54; ≥54) to estimate the direct age-standardized prevalence rates.^45^ We estimated the 95% confidence interval (CI) based on a normal distribution. Finally, we measured the annual differences in age-standardized prevalence rates of specific mental disorders. As a sensitivity analysis, we presented the annual prevalence of specific mental disorders for the year of first OUD diagnosis, and age-standardized incidence rates per 100,000 population (overall population and sex-stratified). All analyses were conducted using SAS 9.4^46^ and R 4.3.0.^47^

## Results

A total of 73,855 individuals aged ≥18 years (female 40.6%, median age 36) had an OUD and concurrent mental disorder between 2013 and 2021 in BC, Canada. Among individuals with OUD and concurrent mental disorders, over 70% have ever received income assistance, 12.5% lived in rural BC, 5.9% were incarcerated, and 7.2% had unstable housing within 12 months of initial indication of OUD (Table 1).

**Table 1.** Descriptive Characteristics of People with Opioid Use Disorder and a Concurrent Mental Disorder, British Columbia, Canada, 2013–2021.

Characteristics, *N* (%)	Total	OUD indication before MD	OUD indication after MD^a^
Number of individuals^b^	73,855 (100%)	10,777 (14.6)	63,078 (85.4)
Demographics			
Female	29,959 (40.6)	2,936 (27.2)	27,023 (42.8)
Age as of first indication of OUD			
18-24 years	9,438 (12.8)	1,164 (10.8)	8,274 (13.1)
25-34 years	19,278 (26.1)	2,926 (27.2)	16,352 (25.9)
35-44 years	16,275 (22.0)	2,729 (25.3)	13,546 (21.5)
45-54 years	14,327 (19.4)	2,265 (21.0)	12,062 (19.1)
≥55 years	14,537 (19.7)	1,693 (15.7)	12,844 (20.4)
Region of residency: rural^c^	9,247 (12.5)	1,036 (9.6)	8,211 (13.0)
Receipt of income assistance ever	54,835 (74.2)	8,431 (78.2)	46,404 (73.6)
Unstable housing as of first date of OUD indication			
Never or >5 years prior	64,062 (86.7)	9,554 (88.7)	54,508 (86.4)
Within 1-5 years prior	4,460 (6.0)	617 (5.7)	3,843 (6.1)
Within 1 year prior	5,333 (7.2)	606 (5.6)	4,727 (7.5)
Incarcerated as of first date of OUD indication			
Never or >1 year prior	66,591 (90.2)	9,633 (89.4)	56,958 (90.3)
Within 1 year prior	4,328 (5.9)	731 (6.8)	3,597 (5.7)
Current	2,936 (4.0)	413 (3.8)	2,523 (4.0)
Specific mental disorders as of end of follow-up			
Anxiety, stress, and adjustment disorder	67,718 (91.7)	8,894 (82.5)	58,824 (93.3)
Depression	54,327 (73.6)	6,086 (56.5)	48,241 (76.5)
Bipolar disorder	26,037 (35.3)	2,069 (19.2)	23,968 (38.0)
Schizophrenia spectrum disorder	15,031 (20.4)	1,608 (14.9)	13,423 (21.3)
Personality disorder	14,435 (19.5)	1,085 (10.1)	13,350 (21.2)
Attention-deficit/hyperactivity disorder	7,738 (10.5)	528 (4.9)	7,210 (11.4)
Developmental disorders	1,071 (1.5)	41 (0.4)	1,030 (1.6)
Other comorbidities as of end of follow-up			
Alcohol use disorder	30,077 (40.7)	3,588 (33.3)	26,489 (42.0)
Nonopioid nonalcohol SUD	62,117 (84.1)	9,121 (84.6)	52,996 (84.0)
Noncancer chronic pain	59,578 (80.7)	7,666 (71.1)	51,912 (82.3)
Hepatitis C virus	10,555 (14.3)	2,594 (24.1)	7,961 (12.6)
HIV/AIDS	2,689 (3.6)	691 (6.4)	1,998 (3.2)
Treatment history as of end of follow-up			
Years since OUD diagnosis, *N* (%)			
<5	28,990 (39.3)	2,043 (19.0)	26,947 (42.7)
5-9	18,505 (25.1)	2,109 (19.6)	16,396 (26.0)
10+	26,360 (35.7)	6,625 (61.5)	19,735 (31.3)
OAT history, *N* (%)			
Ever OAT	49,443 (66.9)	9,457 (87.8)	39,986 (63.4)
Cumulative months on OAT^d^, median (Q1, Q3)	30 (7, 77)	25 (6, 65)	61 (20, 150)
Buprenorphine/naloxone receipt ever	27,905 (37.8)	4,204 (39.0)	23,701 (37.6)
Methadone receipt ever	39,019 (52.8)	8,579 (79.6)	30,440 (48.3)
Other forms of OAT receipt ever	9,424 (12.8)	1,755 (16.3)	7,669 (12.2)
Currently engaged in OAT	19,380 (26.2)	4,003 (37.1)	15,377 (24.4)
Years since mental disorder diagnosis, *N* (%)			
<5	6,410 (8.7)	3,084 (28.6)	3,326 (5.3)
5 to 9	9,066 (12.3)	2,191 (20.3)	6,875 (10.9)
10+	58,379 (79.0)	5,502 (51.1)	52,877 (83.8)
Psychotropic medication prescription			
Antipsychotics			
Number of people with any receipt	30,910 (49.0)	4,873 (45.2)	35,783 (48.5)
cumulative days/person, median (Q1, Q3)	284 (51, 1164)	223 (40, 990)	297 (55, 1194)
Antidepressants			
Number of people with any receipt	44,465 (70.5)	7,228 (67.1)	51,693 (70.0)
Cumulative days/person, median (Q1, Q3)	518 (125, 1630)	355 (90, 1217)	548 (134, 1703)
Antianxiety			
Number of people with any receipt	27,660 (43.9)	3,891 (36.1)	31,551 (42.7)
Cumulative days/person, median (Q1, Q3)	72 (13, 556)	59 (10, 541)	75 (13, 559)
Mood stabilizers			
Number of people with any receipt	27,842 (44.1)	3,930 (36.5)	31,772 (43.0)
Cumulative days/person, median (Q1, Q3)	291 (60, 1080)	215 (46, 869)	301 (60, 1111)
Stimulants			
Number of people with any receipt	6,381 (10.1)	936 (8.7)	7,317 (9.9)
Cumulative days/person, median (Q1, Q3)	213 (59, 656)	209.5 (60, 563)	214 (58, 668)
Acute care visits, total visits/person (median [Q1, Q3])			
All-cause hospitalization	1 (0, 4)	1 (0, 3)	1 (0, 4)
Psychiatric	0 (0, 0)	0 (0, 0)	0 (0, 0)
Drug-related	0 (0, 0)	0 (0, 0)	0 (0, 0)
Hospital readmission within 30 days, *N* (%)^e^	14,727 (30.1)	1,870 (3.8)	12,857 (26.3)
All-cause emergency department visit	4 (1, 10)	4 (1, 11)	3 (1, 10)
Psychiatric	0 (0, 0)	0 (0, 0)	0 (0, 0)
Drug-related	0 (0, 1)	0 (0, 1)	0 (0, 1)
Emergency department readmission within 30 days, *N* (%)^e^	36,385 (63.3)	5,526 (9.6)	30,859 (53.7)
Non-OAT outpatient care visits, total visits/person (median [Q1, Q3])			
All-cause	202 (64, 466)	322 (121, 646)	187 (57, 433)
Psychiatric	4 (1, 13)	4 (1, 11)	4 (0, 14)
Drug-related	13 (0, 142)	83 (3, 285)	9 (0, 118)
All-cause mortality	13,203 (17.9)	1,608 (14.9)	11,595 (18.4)
Lost to follow-up^f^	1,306 (1.8)	305 (2.8)	1,001 (1.6)

Abbreviations: MD = mental disorder; OAT = opioid agonist treatment; OUD = opioid use disorder; SUD = substance use disorder.

^a^ Including the people who were diagnosed with both conditions on the same day.

^b^ Any indication of concurrent OUD and MD between January 01, 1996 and August 31, 2021, and alive and in the study follow-up between January 01, 2013 and August 31, 2021.

^c^ Rural is defined based on population size by local health authority (LHA), details available at https://www2.gov.bc.ca/assets/gov/health/about-bc-s-health-care-system/health-priorites/geographic-service-areas.docx. Rural (LHA population <40,000)/urban (LHA population ≥ 40,000).

^d^ Summary measures among the people who were ever on OAT between 1996 and 2021.

^e^ Among individuals with any episodes of care discharged during study follow-up.

^f^ No healthcare records for 66 months.

Anxiety, stress, and adjustment disorders were the most prevalent (91.7%) mental disorders followed by depression (73.6%), bipolar disorder (35.3%), schizophrenia spectrum disorders (20.4%), and personality disorders (19.5%) (Table 1). Prevalence of multiple mental disorders was also notable (eTable 5, eFigure 1 in the Supplemental Material). In addition to mental disorders the population experienced a high prevalence of other comorbidities including alcohol use disorder (40.7%), nonopioid nonalcohol substance use disorders (84.1%), and chronic pain (80.7%). Within the study population, 67% had ever received OAT including both methadone (52.8%) and buprenorphine/naloxone (37.8%), with a median treatment duration of 30 months (IQR 7, 77). In addition to OAT, receipt of antidepressant (70.5%) prescriptions was the most common followed by antipsychotics (49.0%) and mood stabilizers (44.1%).

The majority (85.4%) of the population experienced their first OUD indication after a mental disorder diagnosis (25.9% were between 25 and 34 years of age at first OUD indication). A higher prevalence of specific mental disorders was observed among this subgroup (*n* = 63,078), when compared to individuals who experienced an OUD indication before a mental disorder diagnosis (*n* = 10,777). Among people with an OUD indication after a mental disorder diagnosis, 63.4% ever received OAT with a median treatment duration of 61 months (IQR 20, 150). Individuals with an OUD indication before a mental disorder diagnosis had greater prevalence of ever receiving OAT (87.8%) but fewer cumulative months on treatment (median 25; IQR 6, 65). All-cause mortality was highest among people with an OUD indication after a mental disorder diagnosis (18.4%). Additional details describing the study population are available in Table 1.

### Annual Prevalence

**Figure 1.**
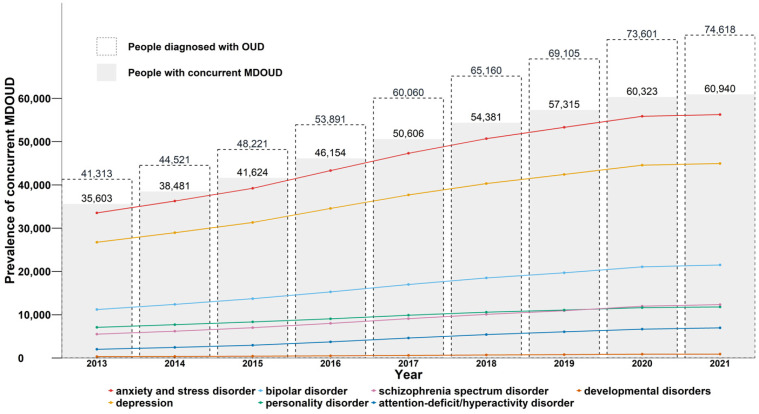
Annual prevalence^a^ of mental disorders among people with opioid use disorder in British Columbia, 2013-2021.^b^

^a^ People who were in the follow-up within the calendar year and diagnosed with opioid use disorder and any of the specific mental disorders ever before the end of the calendar year.

^b^ End of calendar year is August 31, 2021 for the year 2021.

Abbreviations: OUD = opioid use disorder; MDOUD = mental disorder and opioid use disorder.

From 2013 to 2021 the prevalence of any mental disorder among people with OUD and concurrent mental disorders increased by 71% from 35,603 to 60,940, with an average annual percent difference of 7.0% (Table 2, Figure 1). We highlighted a consistently high proportion of people with anxiety and depressive disorders throughout the study period. As of 2021, 92.3% and 73.8% of people were diagnosed with anxiety-related disorders or depression, respectively, and these mental disorders remained the most prevalent codiagnoses among people with OUD over the study period. Concurrently the proportion of people with bipolar disorder rose from 31.5% in 2013 to 35.3% in 2021, while remaining the third most prevalent disorder in each time period. The prevalence of personality disorders (19.9% to 19.4%), and developmental disorders (0.9% to 1.5%) remained relatively stable between 2013 and 2021. In contrast, schizophrenia spectrum disorders and ADHD demonstrated approximately a 120% and 240% increase, respectively, in period prevalence from 2013 to 2021, with the greatest annual percent change occurring between 2014 and 2018. Figure 1 shows the graphical representation of the change in prevalence among individuals with OUD and a concurrent mental disorder. Table 2 highlights the proportion of people with specific mental disorders among people with OUD and a concurrent mental disorder and the annual percent difference from 2013 to 2021.

**Table 2.** Annual Prevalence^a^ of Mental Disorders among People with Opioid Use Disorder and a Concurrent Mental Disorder in British Columbia, 2013 to 2021.^b^

Mental disorders	Description	2013	2014	2015	2016	2017	2018	2019	2020	2021
Any	Period prevalence	35,603	38,481	41,624	46,154	50,606	54,381	57,315	60,323	60,940
	Annual percentage difference		8.1	8.2	10.9	9.6	7.5	5.4	5.2	1.0
Anxiety, stress, and adjustment disorder	Period prevalence	33,530	36,284	39,227	43,314	47,317	50,701	53,323	55,865	56,273
	Annual percentage difference		8.2	8.1	10.4	9.2	7.2	5.2	4.8	0.7
	Proportion among people with concurrent MDOUD, %	94.2	94.3	94.2	93.8	93.5	93.2	93.0	92.6	92.3
Depression	Period prevalence	26,763	28,965	31,334	34,578	37,695	40,320	42,428	44,576	44,947
	Annual percentage difference		8.2	8.2	10.4	9.0	7.0	5.2	5.1	0.8
	Proportion among people with concurrent MDOUD, %	75.2	75.3	75.3	74.9	74.5	74.1	74.0	73.9	73.8
Bipolar disorder	Period prevalence	11,202	12,404	13,709	15,281	16,988	18,502	19,702	21,066	21,514
	Annual percentage difference		10.7	10.5	11.5	11.2	8.9	6.5	6.9	2.1
	Proportion among people with concurrent MDOUD, %	31.5	32.2	32.9	33.1	33.6	34.0	34.4	34.9	35.3
Schizophrenia spectrum disorder	Period prevalence	5,520	6,209	7,012	8,002	9,103	10,090	10,934	11,992	12,336
	Annual percentage difference		12.5	12.9	14.1	13.8	10.8	8.4	9.7	2.9
	Proportion among people with concurrent MDOUD, %	15.5	16.1	16.8	17.3	18.0	18.6	19.1	19.9	20.2
Personality disorder	Period prevalence	7,102	7,703	8,356	9,063	9,900	10,593	11,084	11,657	11,801
	Annual percentage difference		8.5	8.5	8.5	9.2	7.0	4.6	5.2	1.2
	Proportion among people with concurrent MDOUD, %	19.9	20.0	20.1	19.6	19.6	19.5	19.3	19.3	19.4
Attention-deficit/hyperactivity disorder	Period prevalence	2,024	2,457	2,962	3,740	4,637	5,432	6,055	6,685	6,973
	Annual percentage difference		21.4	20.6	26.3	24.0	17.1	11.5	10.4	4.3
	Proportion among people with concurrent MDOUD, %	5.7	6.4	7.1	8.1	9.2	10.0	10.6	11.1	11.4
Developmental disorders	Period prevalence	331	385	429	518	621	720	792	887	922
	Annual percentage difference		16.3	11.4	20.7	19.9	15.9	10.0	12.0	3.9
	Proportion among people with concurrent MDOUD, %	0.9	1.0	1.0	1.1	1.2	1.3	1.4	1.5	1.5

Abbreviations: MDOUD = mental disorder and opioid use disorder.

^a^ People who were in the follow-up within the calendar year and diagnosed with opioid use disorder and any of the specific mental disorders ever before the end of the calendar year.

^b^ End of calendar year is August 31, 2021, for the year 2021.

### Age-Standardized Annual Prevalence Rate by Sex

**Figure 2.**
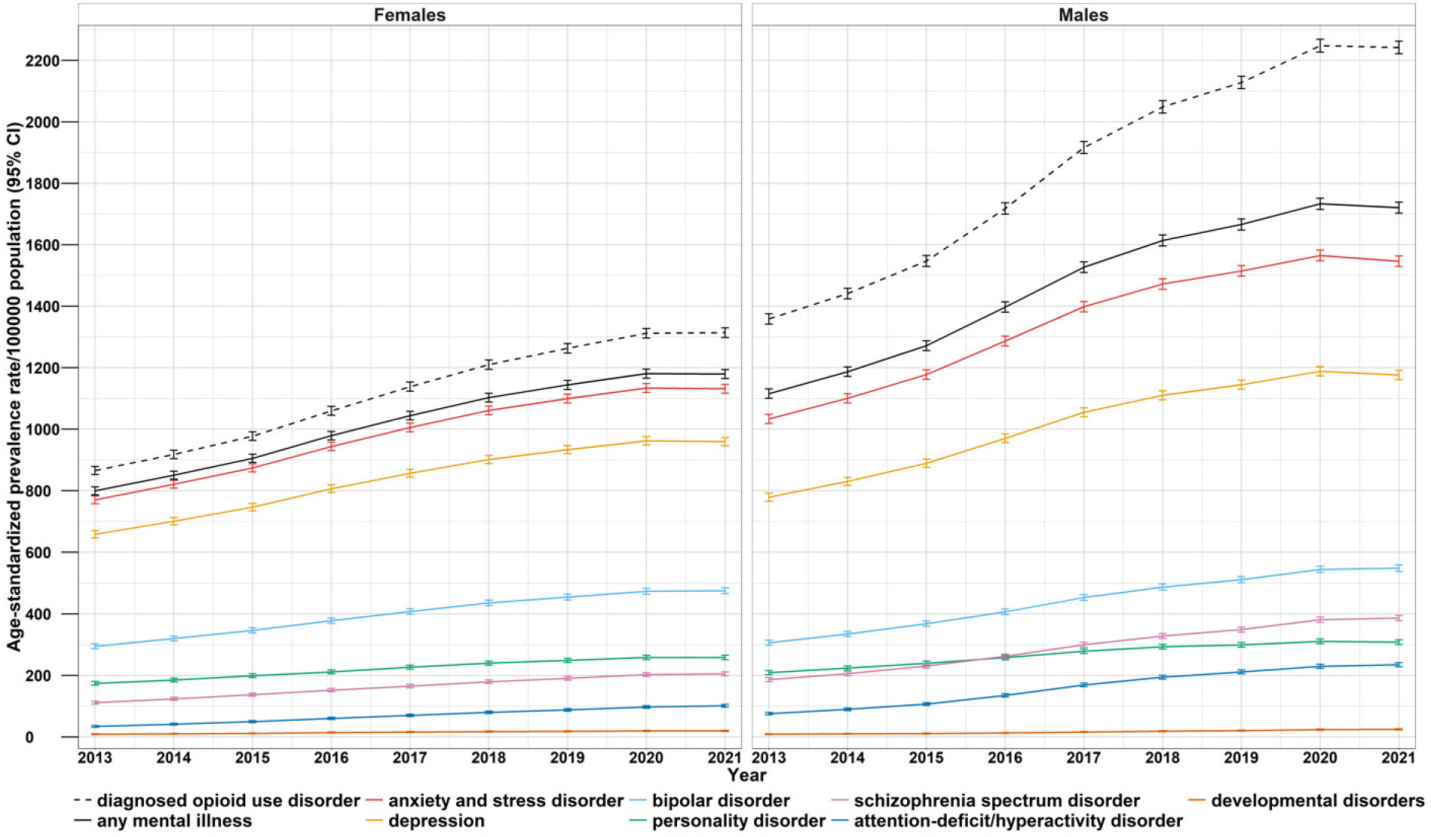
Age-standardized annual prevalence rate (per 100,000 population)^a^ of mental disorders (stratified by sex) and 95% Wald confidence interval (CI) among people with opioid use disorder in British Columbia, 2013-2021.^b^

^a^ Using 2011 Census of Canadian Population data (age grouping: 18-24; 25-34; 35-44; 45-54; ≥54).

^b^ Based on the people who were in the follow-up within the calendar year and diagnosed with opioid use disorder and any of the specific mental disorders ever before the end of the calendar year; end of calendar year is August 31, 2021, for the year 2021.

Annual age-standardized prevalence rates per 100,000 population for specific mental disorders stratified by sex are presented in Figure 2. Continuously increasing age-standardized prevalence was observed for males and females with OUD and any concurrent mental disorder between 2013 and 2021 (age-standardized rate/100,000 population; females: 800, 95% CI [787, 812] to 1179, 95% CI [1165, 1194]; males: 1116, 95% CI [1100, 1131] to 1721, 95% CI [1703, 1739] (Figure 2, eTable 7). Across both sex and year, the two most common mental disorders were anxiety and depression (Figure 2). The least common were ADHD and developmental disorders. Overall, among individuals with OUD and a concurrent mental disorder the prevalence of any mental disorder was higher among males than females and the annual prevalence rate stratified by sex also differed when examining specific mental disorders among this population. The age-standardized annual prevalence rate of anxiety disorders, depression, and bipolar disorder among people with OUD and concurrent mental disorders was higher among males than females (Figure 2). Personality disorders, schizophrenia spectrum disorders, and ADHD were relatively stable over time among females, but greater year-over-year increases were observed for males, most notably for schizophrenia spectrum disorders and ADHD (Figure 2). The annual rate difference for specific mental disorders increased temporally, except for anxiety disorders and depression, which declined among both males and females from 2020 to 2021 (calendar year 2021 ended August 31, 2021), as well as bipolar disorder and personality disorders, which declined among males during the same period (eTable 7).

### Sensitivity Analyses

Annual prevalence of specific mental disorders at first OUD diagnosis (eTable 6 and eFigure 2) and age-standardized annual incidence rates per 100,000 population for specific mental disorders (sex-stratified; eTable 8 and eFigure 3) are presented in the Supplemental Material. The unimodal distribution suggests that the increasing OUD and concurrent mental disorder prevalence between 2016 and 2018 was primarily the result of increasing OUD incidence in the population rather than cumulative disease (eFigure 2). This pattern is further reflected in sex-stratified trends for males (eFigure 3).

## Discussion

This is the first retrospective study to establish prevalence estimates for specific mental disorders among people with OUD and a concurrent mental disorder in BC using population-level administrative data. Our findings demonstrate that concurrent mental disorders are common among this population and highlight an increasing number of individuals diagnosed with OUD and concurrent mental disorders over time. During a nearly 10-year observation period, anxiety and depression remained the most prevalent mental disorders among people with OUD and a concurrent mental disorder in BC. Schizophrenia and bipolar disorder prevalence was persistently high despite impacting a smaller proportion of the population. We found variation in codiagnosis of mental disorders by sex, with prevalence of any mental disorders higher among males than females.

According to prior population-based estimates nearly 60,000 people are diagnosed with OUD in British Columbia, many of whom also experience co-occurring mental illness.^36,43^ Our results demonstrated a growing and consistently high prevalence of any mental disorder among people with OUD and a concurrent mental disorder in BC, supporting and extending previous population-level research in Canada.^48,49^ We observed a high prevalence of both anxiety disorders and depression in our study, surpassing rates previously reported in the United States using national administrative health data.^50,51^ Furthermore, our estimates were consistently two to three times greater for anxiety and depression, and nearly four times greater for bipolar disorder when compared to reported pooled prevalence rates from recent systematic reviews and meta-analyses.^52–54^ Our population-based findings include individuals who are unlikely to consent to participation in prospective cohort studies and those often excluded from randomized controlled trials,^55,56^ which may explain the higher prevalence estimates of mental disorders in our study. Additionally, differences in diagnostic measures or diagnostic groupings for mental disorders may contribute to these variations. Moreover, our results remained consistently higher than those reported in national surveys,^17^ especially among people with severe mental illness.^57,58^ These variations highlight the challenges of relying on self-report data, confirming prior reports of low sensitivity for self-report psychiatric comorbidity among people with OUD,^59^ and underscoring the need for well-defined measurement and reporting mechanisms to ensure accurate population estimates across all mental disorder types. These discrepancies in reported rates are particularly concerning among people with psychotic disorders due to the increased risk for poor outcomes^60^ and increased disease burden.^61^ While schizophrenia is a low-prevalence disorder in the general population (<1%),^62^ prevalence estimates among people with OUD in our study were at minimum 15 times greater than corresponding general population estimates. Furthermore, our results remained elevated even when compared to other OUD populations^52^ and OUD subgroups, such as incarcerated populations,^63^ and people with unstable housing,^64^ with known risk factors for greater prevalence of mental disorders (e.g., criminal histories, polysubstance use, trauma).^65,66^ Our findings highlight a high prevalence of specific mental disorders among people with OUD and concurrent mental disorders and underscore the heterogeneity within the literature potentially underestimating the prevalence, severity, and burden of disease among this population.

Validated algorithms utilizing health administrative data in research have advanced significantly in recent years, bypassing typically lengthy and costly data collection procedures. These algorithms support disease surveillance and risk identification for certain health conditions;^37,67,68^ however, concerns persist about potential misclassification in data collected for nonresearch purposes. A recent systematic review found that while most mental disorders demonstrate moderate reliability in administrative data, well-defined conditions such as schizophrenia yield moderately high predictive values, with the major source of misclassification occurring at the clinical stage.^69^ Our case-finding algorithms utilized multiple population-level administrative health data sources (hospitalization records, emergency department visits, physician billing claims, and prescription data) to maximize sensitivity in identifying individuals with OUD and concurrent mental disorders, an approach validated by prior studies through sensitivity analysis and measurement of diagnostic concordance.^37,39,43,44^ Drug-induced psychosis was excluded as an indicator of any mental disorder in our study since it typically resolves following a period of abstinence, preventing misclassification of temporary conditions as chronic mental disorders. Previous studies employing similar registry-based methodologies support the use of case-finding algorithms as they provide a comprehensive approach to case definition beyond diagnosis alone.^37,43^

Understanding the prevalence and temporal trends of mental disorders among people with OUD can help inform comprehensive clinical and policy actions. Our findings support the need for increased routine screening for mental disorders among people with OUD. Our results demonstrated that the majority of the population experienced a psychiatric illness for 10 or more years and that the first indication of any mental disorder preceded an OUD diagnosis. Routine screening supports early diagnosis and intervention, which are critical because opioid use can heighten the risk of transitioning individuals from a prodromal or subclinical state to a clinically significant mental disorder.^70,71^ In addition to early detection, specific mental disorder prevalence estimates inform targeted mental health treatment approaches. For example, high rates of anxiety and depression in this population suggest treatment efforts should include both psychological treatments (e.g., cognitive behavioural therapy) and pharmacotherapy options.^72^ While prescription medications are the most common form of mental health treatment among OUD populations,^16^ findings from a recent meta-analysis demonstrated that the addition of psychotherapy was associated with a greater reduction of depressive symptoms, when compared to pharmacotherapy alone.^73,74^ While we are unable to estimate the proportion of people receiving non-pharmacological interventions, a large proportion had a history of psychotropic medication dispensation, most notably antipsychotics and antidepressants in addition to ever receiving OAT. Despite the overwhelming need for managing comorbid mental health concerns among people with OUD, there is no clear guidance on prescribing best practices for specific mental disorders and concurrent OAT.^75–78^ Moreover, effective pharmacotherapeutic treatment options for this population remain limited due to the historical exclusion of individuals with substance use disorders from randomized controlled trials examining psychotropic medications.^56,79^ The paucity of experimental studies is a concern and limits the development of evidence-based treatments for individuals with co-occurring mental and substance use disorders.^80^ Given the increasing prevalence of individuals in BC with both OUD and at least one concurrent mental disorder, our findings emphasize the need for additional evidence-based clinical guidance to assist providers in the effective clinical management of this population. In addition, clinical guidance is also required to address complex factors such as polysubstance use, physical health comorbidities, and other social vulnerabilities which create challenges in treatment and diagnosis among this population. Our results confirm that people with OUD and concurrent mental disorders experience several competing health and social comorbidities, and support calls for coordinated, integrated, and low-barrier services that increase access to evidence-based concurrent treatment.^81–85^ Transitioning away from single-disorder and siloed services and moving toward implementation of novel approaches to treatment are essential. Current evidence highlights the emerging use of telemedicine and mobile applications,^86–89^ stepped-care psychotherapy frameworks,^90^ and experimental protocols for collaborative care models which directly account for and address psychiatric comorbidities among OUD populations.^91,92^ Efforts to address psychiatric comorbidity among people with OUD should be prioritized, as they have the potential to reduce the disease burden by decreasing the severity of psychiatric symptoms, increasing quality of life,^93^ and reducing the risk of mortality.^94^ Effective treatment approaches for mental disorders exist; however, access and implementation within OUD populations remain limited and lack clear evidence-based recommendations. It will be critical for future research to explore and evaluate effective pharmacological treatment options and behavioural health models that address the diverse and complex needs of this population.

Our findings also shed light on observed sex differences in the prevalence of mental disorders among people with OUD. Prevalence rates of any mental disorder were uniformly higher in males when compared to females, despite prior research demonstrating greater risk for psychiatric comorbidity among females with OUD.^95^ Furthermore, our findings underscore a marked sex difference in the prevalence of severe mental illnesses, such as schizophrenia and bipolar disorder, with males exhibiting higher rates—a trend documented within the literature.^96^ These sex-specific variations emphasize the need for both research and intervention strategies to account for the unique differences in codiagnoses across distinct demographic groups.

While our study has several strengths, including the use of validated algorithms and population-level administrative data spanning nearly a decade, there are some limitations to consider. This descriptive analysis relies on administrative data which is limited by variability in diagnostic practices among nonspecialized physicians and changes in diagnostic coding systems. Increased recognition of mental health conditions over time may influence observed trends. Also, these datasets exclude health care services and deaths outside of BC, Canada. Our data do not capture individuals presenting with subclinical symptoms falling below the threshold for an OUD. With respect to coding errors, we used ICD codes to attribute OUD and specific mental disorders. While hospitalizations and emergency department data are subject to standardized national-level data quality control annually^97^ and are generally reliable, three-digit ICD-9 codes in physician billings are more reliable than four- or higher-digit codes.^98^ However, this only affects attribution of OUD (304.x [0,7], 305.5, 965.0, E850.0), depression (300.4), or anxiety (300,300.x [0-3, 5-9]) using physician billings. Otherwise, misclassification of bipolar disorder or schizophrenia spectrum disorders in earlier episodes is likely due to overlapping symptoms. To minimize misclassification, we applied a validated case-finding algorithm which required at least three Medical Services Plan records for OUD identification.^43^ In addition to accounting for early and recurrent episodes of bipolar disorder or schizophrenia spectrum disorder, we used a case-finding algorithm validated by the British Columbia Chronic Disease Registries.^89,90^ Furthermore, specialist mental health (i.e., psychologists and/or social workers) service visits were not available in our linked data, thus potentially leading to an underestimate of the prevalence of mental health conditions within the population of people with OUD. Additionally, tertiary psychiatric care is not included, limiting the representation of mental health services.

## Conclusions

Our study shows a persistently high and increasing prevalence of mental disorders among people with OUD and a concurrent mental disorder in BC. Our results provide a population-level estimate of the evolving rates of mental disorders over time and offer valuable information for designing timely and responsive public health and policy interventions. Importantly, our findings emphasize the need for access to evidence-based treatment and support for people experiencing OUD and concurrent mental illness, and warrant replication in other jurisdictions.

## Supplemental Material

sj-docx-1-cpa-10.1177_07067437251347150 - Supplemental material for Prevalence and Temporal Trends of Mental Disorders in Persons with Opioid Use Disorder and Concurrent Mental Disorders in British Columbia, Canada, Using Population-Level Administrative Data, 2013 to 2021: Prévalence et tendances temporelles des troubles mentaux chez les personnes souffrant d’un trouble lié à la consommation d’opioïdes et de troubles mentaux concomitants en Colombie-Britannique, au Canada, à partir de données administratives au niveau de la population, entre 2013 et 2021 Supplemental material, sj-docx-1-cpa-10.1177_07067437251347150 for Prevalence and Temporal Trends of Mental Disorders in Persons with Opioid Use Disorder and Concurrent Mental Disorders in British Columbia, Canada, Using Population-Level Administrative Data, 2013 to 2021: Prévalence et tendances temporelles des troubles mentaux chez les personnes souffrant d’un trouble lié à la consommation d’opioïdes et de troubles mentaux concomitants en Colombie-Britannique, au Canada, à partir de données administratives au niveau de la population, entre 2013 et 2021 by Angela Russolillo, Fahmida Homayra and Bohdan Nosyk in The Canadian Journal of Psychiatry
