# Supplementary material for: Prevalence and Temporal Trends of Mental Disorders in Persons with Opioid Use Disorder and Concurrent Mental Disorders in British Columbia, Canada, Using Population-Level Administrative Data, 2013 to 2021: Prévalence et tendances temporelles des troubles mentaux chez les personnes souffrant d’un trouble lié à la consommation d’opioïdes et de troubles mentaux concomitants en Colombie-Britannique, au Canada, à partir de données administratives au niveau de la population, entre 2013 et 2021
Source: Can J Psychiatry. 2025 Jun 9;70(9):667–80. doi: 10.1177/07067437251347150 (PMC12152009; doi:10.1177/07067437251347150)
Supplement: sj-docx-1-cpa-10.1177_07067437251347150 - Supplemental material for Prevalence and Temporal Trends of Mental Disorders in Persons with Opioid Use Disorder and Concurrent Mental Disorders in British Columbia, Canada, Using Population-Level Administrative Data, 2013 to 2021: Prévalence et tendances te [file sj-docx-1-cpa-10.1177_07067437251347150.docx]

***Supplementary Material:* Prevalence and Temporal Trends of Mental Disorders in Persons with Opioid Use Disorder and Concurrent Mental Disorders in British Columbia, Canada Using Population-level Administrative Data, 2013 to 2021**

Authors: Angela Russolillo, PhD^1, 2, 3^ Fahmida Homayra, MSc,^1,4^ Bohdan Nosyk, PhD ^1,5^

**Affiliations:**

1. Centre for Advancing Health Outcomes, Vancouver, BC, Canada
2. School of Nursing, University of British Columbia, Vancouver, BC, Canada
3. Providence Health Care, Mental Health Program, Vancouver, BC, Canada
4. Centre for Cardiovascular Innovation, Department of Medicine, University of British Columbia, Vancouver, BC, Canada
5. Faculty of Health Sciences, Simon Fraser University, Burnaby, BC, Canada

**Corresponding author:**

Bohdan Nosyk, PhD

Simon Fraser University

Faculty of Health Sciences

8888 University Drive, Burnaby, BC, Canada,V5A 1S6

bohdan_nosyk @sfu.ca

1 778 782-9667

**Disclaimer:** All inferences, opinions, and conclusions drawn in this material are those of the author(s), and do not reflect the opinions or policies of the Data Steward(s).

**Funding Source** Health Canada Substance Use and Addictions Program award no. 1819-HQ-000036 and NIH/NIDA RO1-DA050629.

**Role of Funder** The funding source was independent of the design of this study and did not have any role during its execution, analyses, interpretation of the data, writing, or decision to submit results. All authors had full access to the results in the study and take responsibility for the integrity of the data and accuracy of analysis.

**Conflicts** None

**List of Tables and Figures**

1. eTable 1: Case finding algorithm for opioid use disorder
2. eTable 2: Case finding algorithm for specific mental disorders
3. eTable 3: Case finding algorithm for comorbidities
4. eTable 4: List of psychotropic medications
5. eTable 5: Combination of different mental disorders among people with opioid use disorder and concurrent mental disorders in BC, 2013-2021^a^
6. eTable 6: Annual incidence^a^ of people with opioid use disorder (first indication) and concurrent mental disorders, 2013-2021^b^, British Columbia, Canada
7. eTable 7: Age-standardized prevalence rates^a^ per 100,000 population by specific mental disorders (sex-stratified).
8. eTable 8: Age-standardized incidence rates^a^ per 100,000 population by specific mental disorders (sex-stratified)
9. eFigure 1: Combination of different mental disorders among the people with opioid use disorder and concurrent mental disorders in British Columbia, 2013-2021^a^
10. eFigure 2: Annual incidence ^a^ of individuals with opioid use disorder (first indication) and concurrent mental disorders in British Columbia, 2013-2021^b^
11. eFigure 3: Age-standardized Annual incidencea rate (Per 100,000 Population) among people with opioid use disorder (first indication) and concurrent mental disorders (stratified by sex) and 95% Wald Confidence interval (CI) in British Columbia, 2013-2021^b^

**eTable 1: Case finding algorithm ^a^ for opioid use disorder**

| **Description** | **Code number** | **Database** |
| --- | --- | --- |
| Drug identification number/Product identification number (DIN/PIN) for methadone | 999792, 999793, 66999990, 66999991, 66999992, 66999993, 66999997, 66999998, 66999999, 67000000, 67000001, 67000002, 67000003, 67000004, 67000005, 67000006, 67000007, 67000008, 67000009,67000010, 67000011, 67000012, 67000013, 67000014, 67000015, 67000016, 67000017, 67000018, 67000019, 67000020 | Pharmanet |
| DIN/PIN for buprenorphine/naloxone | 2295695, 2295709, 2408090, 2408104, 2424851, 2424878, 2453908, 2453916, 2468085, 2468093, 2502313, 2502321, 2502348, 2502356, 2517175, 2517183 |  |
| DIN/PIN for slow-release morphine (Kadian) | 22123349, 22123346, 22123347, 22123348 |  |
| DIN/PIN for injectable OAT^†^ | 2146126, 22123340, 22123357, 2469413, 66123367 |  |
| DIN/PIN for t-IOAT (Hydromorphone) | 786543, 885428 |  |
| Fee items related to OAT | 39, 15039,13013,13014,36521 | MSP |
| International classification of Diseases (ICD) 9 | 304.0, 304.7, 305.5, 965.0, E850.0 | MSP |
| International classification of Diseases (ICD) 10-CA | F11, T40.0-T40.4, T40.6 | DAD/NACRS/Vital statistics |
|  | Indication of opioid use as a risk for pregnancy | BCPDR |

Abbreviations: BCPDR: British Columbia Perinatal Data Registry, DAD: Discharge abstract database (hospitalization), MSP: Medical services plan (physician billing), NACRS: National ambulatory care reporting system (emergency department visit), OAT: Opioid agonist treatment

1. To minimize misclassification due to errors in the coding of physician billing records, we applied a case-finding algorithm based on the presence of at least 1 OAT receipt, hospitalization, emergency department visit, BCPDR record, more than 3 physician billing records, or oud-related death record.

**eTable 2: Case finding algorithm for specific mental disorders**

| Mental disorders | ICD 9 ^a^ | ICD 10-CA^b^ | Case finding algorithm ^c^ |
| --- | --- | --- | --- |
| Anxiety, stress and adjustment disorder | 300, 300.0-300.3, 300.5 -300.9, 308, 309 | F40, F41, F42, F48 | ≥1 hospitalization, or ≥ 1 emergency department visit, or ≥1 perinatal record, or ≥ 3 physician-billing records, or death records. |
| Depression | 300.4, 311 | F32, F33, F34.1 | ≥1 hospitalization, ≥1 emergency department visit, or ≥2 physician visits in one year |
| Bipolar disorder | 296 | F30, F31, F34.0, F34.8, F34.9, F38, F39 | ≥1 hospitalization, ≥1 emergency department visit, or ≥2 physician visits in one year |
| Schizophrenia spectrum disorder | 295, 297, 298 | F20, F21, F22, F23, F24, F25, F28, F29 | ≥1 hospitalization, ≥1 emergency department visit, or ≥2 physician visits at least 30 days apart in two years |
| Personality disorder | 301 | F60, F61, F69 | ≥1 hospitalization, or ≥1 emergency department visit, or ≥3 physician-billing records, or death records. |
| Attention-deficit/hyperactivity disorder | 314 | F90 | ≥1 hospitalization, or ≥1 emergency department visit, ≥3 physician-billing records, or death records. |
| Developmental disorders | 317, 318, 319, 760.71 | F70, F71, F72, F73, F78, F79, Q860 | ≥1 hospitalization, or ≥1 emergency department visit, or ≥3 physician-billing records, or death records. |

Abbreviations: ICD: International classification of Diseases.

1. Source database: Medical services plan (physician billing);
2. Source databases: DAD: Discharge abstract database (hospitalization), NACRS: National ambulatory care reporting system (emergency department visit), and BC Vital statistics;
3. To minimize misclassification due to errors in the coding of physician billing records we applied case finding algorithms.

**eTable 3: Case finding algorithm ^a^ for comorbidities**

| Diseases | Diagnostic code | References |
| --- | --- | --- |
| HIV | ICD-9 from DAD and MSP: 042‐044, 079.53, 795.8, V08; ICD-10 from DAD and NACRS: B20‐B24, B97.35, F02.4, O98.7, Z21; MSP fee item: 13015, 13105, 33645, 36370 | (1) |
| Hepatitis C virus | ICD-9 from DAD and MSP: 070.4, 070.5, 070.7; ICD-10 from DAD and NACRS: B17.1, B18.2, B19.2; AHFS category: 8:18.40 | (2), (3), (4) |
| Alcohol use disorder | ICD-9 from DAD and MSP: 291, 303, 305.0, 357.5, 425.5, 535.3, 571.0-571.3, 655.4, V65.42; ICD-10 from DAD and NACRS: F10, Z50.2, Z71.4, Z72.1, G31.2, G62.1, G72.1, I42.6, K29.2, K70, K86.0, O35.4; DIN: 2293269, 2158655, 2213826, 2444275, 2451883, 2534, 2542, 2041375, 2041391, 66124089, 66124085, 66124087; indication of alcohol use disorder during the pregnancy from BCPDR | (5), (6) |
| Substance use disorder ^b^ | ICD-9 from DAD and MSP: 292, 304.x (1-6,8,9), 305.x (2-4,6-9), 648.3, 655.5, 967, 969.x (4,6,7), 970, E851, E852, E853.2, E854.x (1,2,3); ICD-10 from DAD and NACRS: F12-F16, F19, X42, X62, Y12, T40.5, T40.7-T40.9, T42.4, T43.6, Z50.3, Z71.5, Z72.2; indication of substance use during the pregnancy from BCPDR | (7), (5) |
| Non-cancer chronic pain | ICD-9 from DAD and MSP: 338.2, 338.4, 307.80, 307.89, 338.0, 719.41, 719.45-719.47, 719.49, 720.0, 720.2, 720.9, 721.0-721.4, 721.6, 721.8, 721.9, 722, 723.0, 723.1, 723.3-723.9, 724.0-724.6, 724.70, 724.79, 724.8, 724.9, 729.0-729.2, 729.4, 729.5, 350, 352-357, 344.0, 344.1, 997.0, 733.0, 733.7, 733.9, 781; ICD-10 from DAD and NACRS: F45.4, G89.0, G89.2, G89.4, M08.1, M25.50, M25.51, M25.55-M25.57, M43.2-M43.6, M45, M46.1, M46.3, M46.4, M46.9, M47, M48.0, M48.1, M48.8, M48.9, M50.8, M50.9, M51, M53.1-M53.3, M53.8, M53.9, M54, M60.8, M60.9, M63.3, M79.0-M79.2, M79.6, M79.7, M96.1, G50, G52-G64, G82, G97, M89, R29 | (8) |

Abbreviations*:* ICD: International Classification of Diseases; DAD: Discharge Abstract Database (records of hospitalizations); NACRS: National Ambulatory Care Reporting System (records of emergency visits); MSP: Medical Service Plan (physician billing records); DIN: drug identification number; BCPDR: British Columbia Perinatal data registry.

1. To minimize misclassification due to errors in the coding of physician billing records, we applied a case-finding algorithm based on the presence of at least 1 hospitalization, emergency department visit, perinatal substance use record, more than 3 physician billing records, or medication receipt for alcohol use disorder;
2. Any indication of non-opioid drug use, poisoning (accidental or intentional), or substance use counselling or rehab, excluding alcohol use disorder;

**eTable 4: List of psychotropic medications**

| **Type of Medication** | **American hospital formulary service code in Pharmanet** | **Generic name** |
| --- | --- | --- |
| Antidepressants | 281604 | Amitriptyline Hcl, Amitriptyline Pamoate, Amoxapine, Bupropion Hcl, Citalopram Hydrobromide, Clomipramine Hcl, Desipramine Hcl, Desvenlafaxine Succinate, Doxepin Hcl, Duloxetine Hcl, Escitalopram, Escitalopram Oxalate, Fluoxetine Hcl, Fluvoxamine Maleate, Imipramine Hcl, Levomilnacipran Hcl, Maprotiline Hcl, Mirtazapine, Moclobemide, Nefazodone Hcl, Nortriptyline Hcl, Paroxetine Hcl, Perphenazine/Amitriptyline Hcl, Phenelzine Sulfate, Protriptyline Hcl, Sertraline Hcl, Tranylcypromine Sulfate, Trazodone Hcl, Trimipramine Maleate, Venlafaxine Hcl, Vortioxetine Hydrobromide. |
| Anti-anxiety | 281208, 282408 | Alprazolam, Bromazepam, Chlordiazepoxide Hcl, Chlordiazepoxide/Clidinium Br, Clobazam, Clonazepam,  Diazepam, Diazepam (In Soybean Oil), Flurazepam Hcl, Ketazolam, Lorazepam, Midazolam Hcl, Midazolam Hcl/Pf, Nitrazepam, Oxazepam, Temazepam, Triazolam, Clobazam, Clonazepam, Alprazolam, Bromazepam, Chlordiazepoxide Hcl, Clorazepate Dipotassium, Diazepam, Diazepam (In Soybean Oil), Estazolam, Flurazepam Hcl, Ketazolam, Lorazepam, Midazolam Hcl, Midazolam Hcl/Pf, Nitrazepam, Oxazepam, Temazepam, Triazolam. |
| Antipsychotics | 281608 | Aripiprazole, Asenapine Maleate, Chlorpromazine Hcl, Clozapine, Flupentixol Decanoate, Flupentixol Di-Hcl, Fluphenazine Decanoate, Fluphenazine Enanthate, Fluphenazine Hcl, Fluspirilene, Haloperidol, Haloperidol Decanoate, Haloperidol Lactate, Loxapine Hcl, Loxapine Succinate, Lurasidone Hcl, Mesoridazine Besylate, Methotrimeprazine Hcl, Methotrimeprazine Maleate, Olanzapine, Paliperidone, Paliperidone Palmitate, Periciazine, Perphenazine, Pimozide, Pipotiazine Palmitate, Quetiapine Fumarate, Risperidone, Risperidone Microspheres, Thioproperazine Mesylate, Thioridazine Hcl, Thiothixene, Trifluoperazine Hcl, Ziprasidone Hcl, Zuclopenthixol Acetate, Zuclopenthixol Decanoate, Zuclopenthixol Hcl. |
| Mood stabilizers | 281292, 281212,  282800 | Carbamazepine, Divalproex Sodium, Eslicarbazepine Acetate, Gabapentin, Lacosamide, Lamotrigine, Levetiracetam, Lithium Carbonate, Lithium Citrate, Oxcarbazepine, Perampanel, Phenytoin, Phenytoin Sodium, Phenytoin Sodium Extended, Pregabalin, Rufinamide, Topiramate, Valproic Acid, Valproic Acid (As Sodium Salt), Vigabatrin |
| Stimulants | 282004, 282032, 282092 | Dextroamphetamine Sulfate, Methylphenidate Hcl, Dextroamphetamine/Amphetamine, Lisdexamfetamine Dimesylate. Pemoline |

**eTable 5: Combination of different mental disorders among people with opioid use disorder and concurrent mental disorders in BC, 2013-2021^a^**

|  |  | Anxiety, stress and adjustment disorder | Depression | Bipolar disorder | Schizophrenia spectrum disorder | Personality disorder | Attention-deficit/hyperactivity disorder | Developmental disorders |
| --- | --- | --- | --- | --- | --- | --- | --- | --- |
| Anxiety, stress and adjustment disorder | Count | **67,718** | 50,080 | 24,586 | 13,718 | 13,877 | 6,994 | 1,009 |
|  | % | 100 | 73.95 | 36.31 | 20.26 | 20.49 | 10.33 | 1.49 |
| Depression | Count | 50,080 | **54,327** | 23,016 | 12,242 | 13,026 | 5,814 | 901 |
|  | % | 92.18 | 100 | 42.37 | 22.53 | 23.98 | 10.7 | 1.66 |
| Bipolar disorder | Count | 24,586 | 23,016 | **26,037** | 10,543 | 10,012 | 3,556 | 761 |
|  | % | 94.43 | 88.4 | 100 | 40.49 | 38.45 | 13.66 | 2.92 |
| Schizophrenia spectrum disorder | Count | 13,718 | 12,242 | 10,543 | **15,031** | 6,786 | 2,308 | 698 |
|  | % | 91.26 | 81.45 | 70.14 | 100 | 45.15 | 15.35 | 4.64 |
| Personality disorder | Count | 13,877 | 13,026 | 10,012 | 6,786 | **14,435** | 2,294 | 564 |
|  | % | 96.13 | 90.24 | 69.36 | 47.01 | 100 | 15.89 | 3.91 |
| Attention-deficit/hyperactivity disorder | Count | 6,994 | 5,814 | 3,556 | 2,308 | 2,294 | **7,738** | 454 |
|  | % | 90.39 | 75.14 | 45.96 | 29.83 | 29.65 | 100 | 5.87 |
| Developmental disorders | Count | 1,009 | 901 | 761 | 698 | 564 | 454 | **1,071** |
|  | % | 94.21 | 84.13 | 71.06 | 65.17 | 52.66 | 42.39 | 100 |

1. Period prevalence of people with concurrent opioid use disorder and mental disorder between 01/2013 and 08/2021.

**eTable 6: Annual incidence^a^ of people with opioid use disorder (first indication) and concurrent mental disorders, 2013-2021^b^, British Columbia, Canada**

| Mental disorders | Description | 2013 | 2014 | 2015 | 2016 | 2017 | 2018 | 2019 | 2020 | 2021^b^ |
| --- | --- | --- | --- | --- | --- | --- | --- | --- | --- | --- |
| Any | Incidence | 3422 | 3666 | 3914 | 5398 | 5690 | 5228 | 4345 | 4206 | 2433 |
|  | Annual percentage difference | NA | 7.1 | 6.8 | 37.9 | 5.4 | -8.1 | -16.9 | -3.2 | -42.2 |
| Anxiety, stress and adjustment disorder | Incidence | 3120 | 3385 | 3558 | 4823 | 5036 | 4618 | 3827 | 3673 | 2107 |
|  | Annual percentage difference | NA | 8.5 | 5.1 | 35.6 | 4.4 | -8.3 | -17.1 | -4.0 | -42.6 |
|  | Proportion among incident concurrent MDOUD, % | 91.2 | 92.3 | 90.9 | 89.3 | 88.5 | 88.3 | 88.1 | 87.3 | 86.6 |
| Depression | Incidence | 2454 | 2576 | 2726 | 3673 | 3791 | 3476 | 2932 | 2803 | 1601 |
|  | Annual percentage difference | NA | 5.0 | 5.8 | 34.7 | 3.2 | -8.3 | -15.7 | -4.4 | -42.9 |
|  | Proportion among incident concurrent MDOUD, % | 71.7 | 70.3 | 69.6 | 68.0 | 66.6 | 66.5 | 67.5 | 66.6 | 65.8 |
| Bipolar disorder | Incidence | 990 | 1050 | 1196 | 1501 | 1702 | 1639 | 1378 | 1425 | 846 |
|  | Annual percentage difference | NA | 6.1 | 13.9 | 25.5 | 13.4 | -3.7 | -15.9 | 3.4 | -40.6 |
|  | Proportion among incident concurrent MDOUD, % | 28.9 | 28.6 | 30.6 | 27.8 | 29.9 | 31.4 | 31.7 | 33.9 | 34.8 |
| Schizophrenia spectrum disorder | Incidence | 446 | 525 | 598 | 819 | 977 | 959 | 807 | 915 | 526 |
|  | Annual percentage difference | NA | 17.7 | 13.9 | 37.0 | 19.3 | -1.8 | -15.8 | 13.4 | -42.5 |
|  | Proportion among incident concurrent MDOUD, % | 13.0 | 14.3 | 15.3 | 15.2 | 17.2 | 18.3 | 18.6 | 21.8 | 21.6 |
| Personality disorder | Incidence | 473 | 537 | 608 | 750 | 886 | 785 | 642 | 663 | 404 |
|  | Annual percentage difference | NA | 13.5 | 13.2 | 23.4 | 18.1 | -11.4 | -18.2 | 3.3 | -39.1 |
|  | Proportion among incident concurrent MDOUD, % | 13.8 | 14.6 | 15.5 | 13.9 | 15.6 | 15.0 | 14.8 | 15.8 | 16.6 |
| Attention-deficit/hyperactivity disorder | Incidence | 299 | 324 | 395 | 623 | 779 | 657 | 519 | 502 | 302 |
|  | Annual percentage difference | NA | 8.4 | 21.9 | 57.7 | 25.0 | -15.7 | -21.0 | -3.3 | -39.8 |
|  | Proportion among incident concurrent MDOUD, % | 8.7 | 8.8 | 10.1 | 11.5 | 13.7 | 12.6 | 11.9 | 11.9 | 12.4 |
| Developmental disorders | Incidence | 33 | 40 | 34 | 78 | 97 | 85 | 76 | 78 | 46 |
|  | Annual percentage difference | NA | 21.2 | -15.0 | 129.4 | 24.4 | -12.4 | -10.6 | 2.6 | -41.0 |
|  | Proportion among incident concurrent MDOUD, % | 1.0 | 1.1 | 0.9 | 1.4 | 1.7 | 1.6 | 1.7 | 1.9 | 1.9 |

Abbreviations: MDOUD: : individuals with opioid use disorder (OUD) and concurrent mental disorder mental disorders (MD)

NA: not applicable.

1. People who were in the follow up within the calendar year and diagnosed with opioid use disorder for the first time within the calendar year and with a prior mental disorder.
2. End of calendar year is 31 August 2021 for the year 2021.

**eTable 7: Age-standardized prevalence rates^a^ per 100,000 population by specific mental disorders (sex-stratified).**

| Female | | | | | Male | | | |
| --- | --- | --- | --- | --- | --- | --- | --- | --- |
| Year | **Age Standardized Rate per 100,000 Population** | **Lower Confidence Limit** | **Upper Confidence Limit** | **Rate Difference (t+1) - t** | **Age Standardized Rate per 100,000 Population** | **Lower Confidence Limit** | **Upper Confidence Limit** | **Rate Difference (t+1) - t** |
| Any mental disorders | | | | | | | | |
| 2013 | 800 | 787 | 812 | NA | 1116 | 1100 | 1131 | NA |
| 2014 | 850 | 837 | 863 | 51 | 1187 | 1171 | 1203 | 71 |
| 2015 | 905 | 892 | 919 | 55 | 1271 | 1255 | 1288 | 85 |
| 2016 | 979 | 965 | 993 | 74 | 1397 | 1380 | 1414 | 126 |
| 2017 | 1044 | 1030 | 1058 | 65 | 1527 | 1509 | 1544 | 130 |
| 2018 | 1102 | 1088 | 1117 | 59 | 1614 | 1596 | 1632 | 87 |
| 2019 | 1144 | 1129 | 1159 | 41 | 1666 | 1648 | 1684 | 52 |
| 2020 | 1181 | 1166 | 1195 | 37 | 1733 | 1715 | 1752 | 67 |
| 2021 | 1179 | 1165 | 1194 | -1 | 1721 | 1703 | 1739 | -13 |
| Anxiety, stress and adjustment disorder | | | | | | | | |
| 2013 | 770 | 758 | 783 | NA | 1033 | 1019 | 1048 | NA |
| 2014 | 821 | 808 | 834 | 51 | 1100 | 1085 | 1115 | 67 |
| 2015 | 874 | 861 | 887 | 53 | 1178 | 1162 | 1193 | 77 |
| 2016 | 943 | 930 | 957 | 70 | 1287 | 1271 | 1303 | 109 |
| 2017 | 1005 | 991 | 1019 | 62 | 1398 | 1382 | 1415 | 112 |
| 2018 | 1061 | 1047 | 1075 | 56 | 1472 | 1455 | 1489 | 74 |
| 2019 | 1100 | 1085 | 1114 | 39 | 1515 | 1498 | 1532 | 43 |
| 2020 | 1134 | 1119 | 1148 | 34 | 1565 | 1548 | 1583 | 50 |
| 2021 | 1131 | 1117 | 1146 | -2 | 1547 | 1529 | 1564 | -19 |
| Depression | | | | | | | | |
| 2013 | 659 | 647 | 670 | NA | 779 | 766 | 792 | NA |
| 2014 | 701 | 689 | 713 | 42 | 830 | 817 | 843 | 51 |
| 2015 | 746 | 734 | 759 | 46 | 889 | 876 | 903 | 59 |
| 2016 | 806 | 794 | 819 | 60 | 970 | 956 | 984 | 81 |
| 2017 | 857 | 844 | 869 | 50 | 1055 | 1040 | 1069 | 85 |
| 2018 | 901 | 888 | 915 | 45 | 1110 | 1095 | 1125 | 55 |
| 2019 | 933 | 920 | 946 | 32 | 1145 | 1130 | 1160 | 35 |
| 2020 | 962 | 949 | 975 | 29 | 1188 | 1173 | 1203 | 44 |
| 2021 | 959 | 946 | 973 | -3 | 1176 | 1161 | 1191 | -12 |
| Bipolar disorder | | | | | | | | |
| 2013 | 295 | 287 | 302 | NA | 209 | 202 | 215 | NA |
| 2014 | 320 | 312 | 328 | 25 | 224 | 217 | 231 | 15 |
| 2015 | 346 | 338 | 355 | 26 | 239 | 232 | 246 | 15 |
| 2016 | 378 | 369 | 386 | 31 | 258 | 250 | 265 | 18 |
| 2017 | 408 | 399 | 416 | 30 | 279 | 271 | 286 | 21 |
| 2018 | 436 | 426 | 445 | 28 | 293 | 285 | 301 | 14 |
| 2019 | 454 | 445 | 464 | 19 | 299 | 292 | 307 | 6 |
| 2020 | 473 | 464 | 483 | 19 | 311 | 303 | 318 | 11 |
| 2021 | 475 | 466 | 485 | 3 | 308 | 300 | 316 | -3 |
| Schizophrenia spectrum disorder | | | | | | | | |
| 2013 | 111 | 107 | 116 | NA | 186 | 180 | 192 | NA |
| 2014 | 124 | 119 | 129 | 12 | 206 | 199 | 212 | 20 |
| 2015 | 137 | 132 | 143 | 14 | 231 | 224 | 238 | 25 |
| 2016 | 152 | 147 | 158 | 15 | 262 | 255 | 269 | 31 |
| 2017 | 165 | 160 | 171 | 13 | 300 | 292 | 308 | 38 |
| 2018 | 179 | 173 | 185 | 14 | 328 | 320 | 336 | 28 |
| 2019 | 190 | 184 | 196 | 11 | 349 | 341 | 357 | 21 |
| 2020 | 203 | 196 | 209 | 12 | 381 | 372 | 389 | 32 |
| 2021 | 205 | 199 | 211 | 3 | 386 | 378 | 395 | 5 |
| Personality disorder | | | | | | | | |
| 2013 | 174 | 168 | 180 | NA | 209 | 202 | 215 | NA |
| 2014 | 185 | 179 | 191 | 11 | 224 | 217 | 231 | 15 |
| 2015 | 199 | 193 | 205 | 14 | 239 | 232 | 246 | 15 |
| 2016 | 211 | 205 | 218 | 12 | 258 | 250 | 265 | 18 |
| 2017 | 227 | 220 | 233 | 16 | 279 | 271 | 286 | 21 |
| 2018 | 240 | 233 | 247 | 13 | 293 | 285 | 301 | 14 |
| 2019 | 249 | 242 | 255 | 9 | 299 | 292 | 307 | 6 |
| 2020 | 258 | 251 | 265 | 9 | 311 | 303 | 318 | 11 |
| 2021 | 258 | 251 | 265 | 0 | 308 | 300 | 316 | -3 |
| Attention-deficit/hyperactivity disorder | | | | | | | | |
| 2013 | 34 | 32 | 37 | NA | 76 | 72 | 80 | NA |
| 2014 | 41 | 39 | 44 | 7 | 90 | 85 | 94 | 14 |
| 2015 | 50 | 47 | 53 | 8 | 107 | 102 | 112 | 17 |
| 2016 | 60 | 57 | 64 | 10 | 135 | 130 | 140 | 28 |
| 2017 | 70 | 66 | 74 | 10 | 169 | 163 | 175 | 34 |
| 2018 | 80 | 76 | 84 | 10 | 194 | 188 | 200 | 25 |
| 2019 | 88 | 84 | 92 | 8 | 211 | 205 | 218 | 17 |
| 2020 | 97 | 93 | 102 | 9 | 229 | 223 | 236 | 18 |
| 2021 | 101 | 97 | 106 | 4 | 235 | 228 | 241 | 5 |
| Developmental disorders | | | | | | | | |
| 2013 | 9 | 8 | 10 | NA | 9 | 8 | 10 | NA |
| 2014 | 10 | 9 | 12 | 1 | 10 | 9 | 12 | 1 |
| 2015 | 12 | 10 | 13 | 2 | 11 | 10 | 13 | 1 |
| 2016 | 14 | 12 | 16 | 2 | 13 | 11 | 15 | 2 |
| 2017 | 16 | 14 | 18 | 2 | 16 | 14 | 18 | 3 |
| 2018 | 18 | 16 | 19 | 2 | 19 | 17 | 21 | 3 |
| 2019 | 18 | 17 | 20 | 1 | 21 | 19 | 23 | 2 |
| 2020 | 20 | 18 | 22 | 1 | 24 | 22 | 26 | 3 |
| 2021 | 20 | 18 | 22 | 0 | 25 | 23 | 27 | 1 |

Abbreviations: NA: not applicable

1. 2021 rates were measured using data from 01/2021 to 08/2021.

**eTable 8: Age-standardized incidence rates^a^ per 100,000 population by specific mental disorders (sex-stratified)**

| Female | | | | | Male | | | |
| --- | --- | --- | --- | --- | --- | --- | --- | --- |
| Year | **Age Standardized Rate per 100,000 Population** | **Lower Confidence Limit** | **Upper Confidence Limit** | **Rate Difference (t+1) - t** | **Age Standardized Rate per 100,000 Population** | **Lower Confidence Limit** | **Upper Confidence Limit** | **Rate Difference (t+1) - t** |
| Any mental disorders | | | | | | | | |
| 2013 | 83 | 79 | 87 | NA | 101 | 96 | 106 | NA |
| 2014 | 82 | 78 | 86 | -1 | 111 | 107 | 116 | 10 |
| 2015 | 85 | 81 | 90 | 3 | 120 | 115 | 125 | 8 |
| 2016 | 108 | 103 | 112 | 22 | 171 | 165 | 177 | 51 |
| 2017 | 102 | 98 | 107 | -6 | 188 | 181 | 194 | 17 |
| 2018 | 100 | 96 | 105 | -2 | 160 | 154 | 165 | -28 |
| 2019 | 86 | 82 | 90 | -14 | 124 | 120 | 129 | -35 |
| 2020 | 78 | 75 | 82 | -8 | 124 | 119 | 129 | -1 |
| 2021 | 46 | 43 | 49 | -33 | 70 | 67 | 74 | -54 |
| Anxiety, stress and adjustment disorder | | | | | | | | |
| 2013 | 78 | 74 | 82 | NA | 89 | 85 | 94 | NA |
| 2014 | 79 | 75 | 83 | 1 | 100 | 95 | 104 | 10 |
| 2015 | 81 | 77 | 85 | 2 | 106 | 101 | 110 | 6 |
| 2016 | 101 | 96 | 105 | 20 | 148 | 143 | 154 | 43 |
| 2017 | 96 | 92 | 100 | -5 | 160 | 155 | 166 | 12 |
| 2018 | 94 | 90 | 98 | -2 | 136 | 131 | 141 | -24 |
| 2019 | 81 | 77 | 85 | -13 | 105 | 100 | 109 | -31 |
| 2020 | 74 | 70 | 78 | -7 | 103 | 98 | 107 | -2 |
| 2021 | 43 | 40 | 45 | -31 | 58 | 55 | 61 | -45 |
| Depression | | | | | | | | |
| 2013 | 66 | 62 | 69 | NA | 66 | 62 | 69 | NA |
| 2014 | 65 | 61 | 68 | -1 | 71 | 67 | 75 | 5 |
| 2015 | 67 | 63 | 70 | 2 | 76 | 72 | 80 | 5 |
| 2016 | 85 | 80 | 88 | 18 | 105 | 100 | 110 | 29 |
| 2017 | 78 | 74 | 82 | -7 | 115 | 110 | 120 | 10 |
| 2018 | 76 | 72 | 80 | -2 | 97 | 93 | 101 | -18 |
| 2019 | 66 | 62 | 70 | -10 | 76 | 73 | 80 | -21 |
| 2020 | 60 | 56 | 63 | -7 | 76 | 72 | 80 | -1 |
| 2021 | 34 | 32 | 37 | -25 | 43 | 40 | 45 | -33 |
| Bipolar disorder | | | | | | | | |
| 2013 | 12 | 11 | 14 | NA | 25 | 23 | 27 | NA |
| 2014 | 13 | 11 | 15 | 1 | 28 | 26 | 31 | 3 |
| 2015 | 16 | 14 | 17 | 3 | 33 | 30 | 35 | 4 |
| 2016 | 17 | 15 | 18 | 1 | 42 | 39 | 44 | 9 |
| 2017 | 19 | 17 | 21 | 2 | 51 | 48 | 54 | 9 |
| 2018 | 18 | 16 | 20 | -1 | 45 | 42 | 48 | -5 |
| 2019 | 15 | 14 | 17 | -3 | 37 | 35 | 40 | -8 |
| 2020 | 14 | 12 | 16 | -1 | 41 | 38 | 43 | 3 |
| 2021 | 9 | 8 | 10 | -5 | 25 | 22 | 27 | -16 |
| Schizophrenia spectrum disorder | | | | | | | | |
| 2013 | 9 | 7 | 10 | NA | 15 | 13 | 17 | NA |
| 2014 | 10 | 8 | 11 | 1 | 18 | 16 | 20 | 3 |
| 2015 | 11 | 9 | 12 | 1 | 21 | 19 | 23 | 3 |
| 2016 | 13 | 12 | 15 | 2 | 29 | 27 | 32 | 9 |
| 2017 | 13 | 11 | 14 | 0 | 37 | 35 | 40 | 8 |
| 2018 | 15 | 13 | 16 | 2 | 34 | 31 | 36 | -4 |
| 2019 | 13 | 11 | 15 | -2 | 27 | 24 | 29 | -7 |
| 2020 | 12 | 11 | 14 | -1 | 33 | 30 | 35 | 6 |
| 2021 | 8 | 6 | 9 | -4 | 18 | 16 | 19 | -15 |
| Personality disorder | | | | | | | | |
| 2013 | 12 | 11 | 14 | NA | 13 | 12 | 15 | NA |
| 2014 | 13 | 11 | 15 | 1 | 16 | 14 | 17 | 2 |
| 2015 | 16 | 14 | 17 | 3 | 17 | 15 | 18 | 1 |
| 2016 | 17 | 15 | 18 | 1 | 23 | 20 | 25 | 6 |
| 2017 | 19 | 17 | 21 | 2 | 27 | 25 | 29 | 4 |
| 2018 | 18 | 16 | 20 | -1 | 22 | 20 | 24 | -5 |
| 2019 | 15 | 14 | 17 | -3 | 16 | 15 | 18 | -6 |
| 2020 | 14 | 12 | 16 | -1 | 19 | 17 | 21 | 2 |
| 2021 | 9 | 8 | 10 | -5 | 11 | 9 | 12 | -8 |
| Attention-deficit/hyperactivity disorder | | | | | | | | |
| 2013 | 5 | 4 | 6 | NA | 11 | 10 | 13 | NA |
| 2014 | 5 | 4 | 6 | 0 | 12 | 11 | 14 | 1 |
| 2015 | 6 | 5 | 8 | 1 | 15 | 13 | 16 | 2 |
| 2016 | 8 | 6 | 9 | 1 | 25 | 23 | 27 | 10 |
| 2017 | 8 | 7 | 10 | 1 | 32 | 29 | 34 | 7 |
| 2018 | 8 | 7 | 9 | 0 | 25 | 23 | 27 | -7 |
| 2019 | 7 | 5 | 8 | -1 | 19 | 17 | 21 | -6 |
| 2020 | 7 | 5 | 8 | 0 | 18 | 16 | 20 | -1 |
| 2021 | 4 | 3 | 5 | -3 | 11 | 9 | 12 | -7 |
| Developmental disorders | | | | | | | | |
| 2013 | 1 | 1 | 1 | NA | 1 | 0 | 1 | NA |
| 2014 | 1 | 0 | 1 | 0 | 1 | 1 | 2 | 0 |
| 2015 | 1 | 1 | 2 | 0 | 1 | 0 | 1 | 0 |
| 2016 | 2 | 1 | 3 | 1 | 2 | 1 | 3 | 1 |
| 2017 | 2 | 1 | 2 | 0 | 3 | 3 | 4 | 1 |
| 2018 | 2 | 1 | 2 | 0 | 3 | 2 | 3 | -1 |
| 2019 | 1 | 1 | 2 | 0 | 2 | 2 | 3 | 0 |
| 2020 | 1 | 1 | 2 | 0 | 3 | 2 | 3 | 0 |
| 2021 | 1 | 0 | 1 | 0 | 1 | 1 | 2 | -1 |

Abbreviations: NA: not applicable.

1. 2021 rates were measured using data from 01/2021 to 08/20.

**eFigure 1: Combination of different mental disorders among the people with opioid use disorder and concurrent mental disorders in British Columbia, 2013-2021^a^**

**
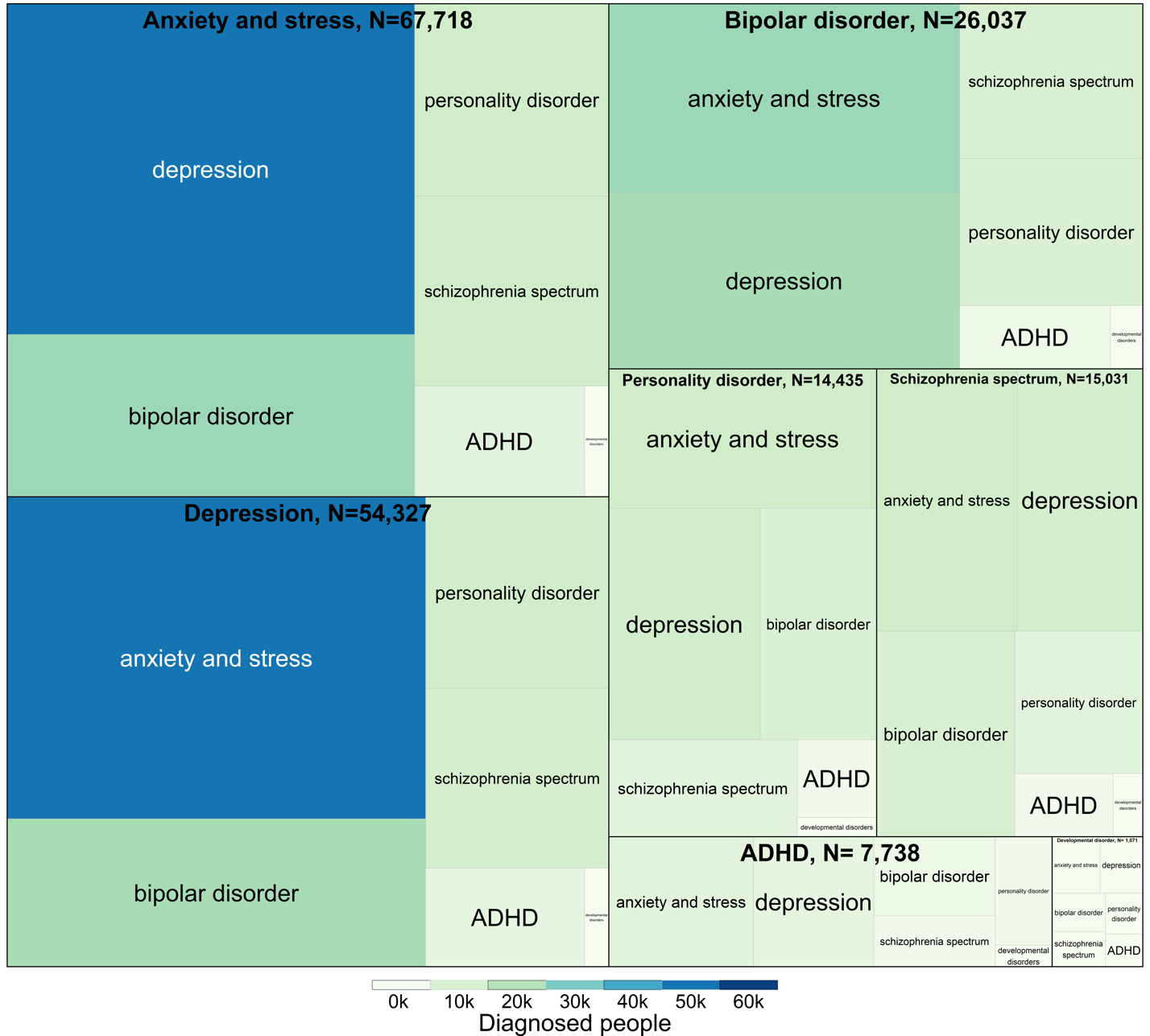
**

Abbreviations: ADHD: attention-deficit/hyperactivity disorder.

1. Point prevalence of people with opioid use disorder and concurrent mental disorder between 01/2013 and 08/2021.

**eFigure 2:** **Annual incidence ^a^ of individuals with opioid use disorder (first indication) and concurrent mental disorders in British Columbia, 2013-2021^b^**


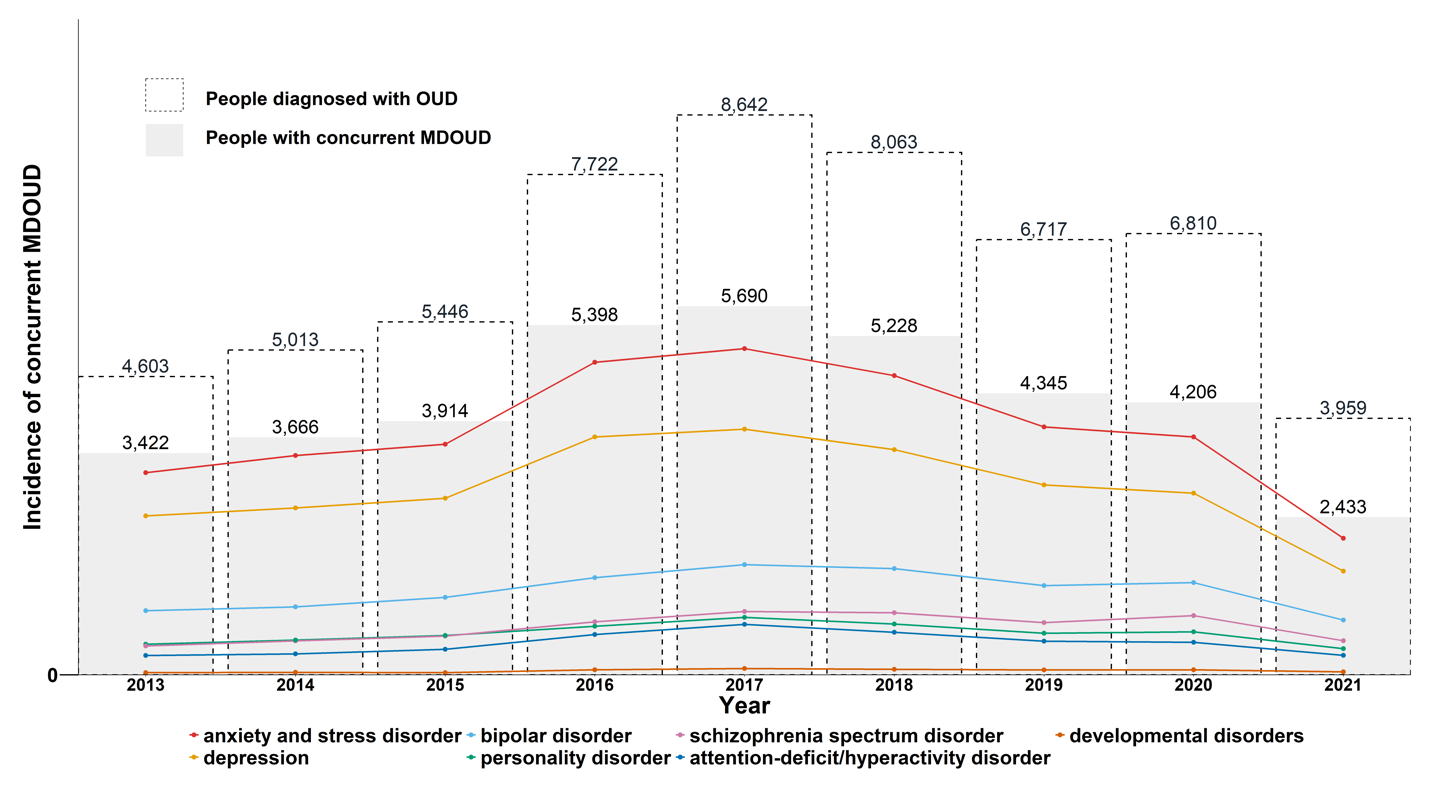


Abbreviations: MDOUD: individuals with opioid use disorder (OUD) and concurrent mental disorder mental disorders (MD)

1. People who were in the follow up within the calendar year and diagnosed with opioid use disorder for the first time withiht the calendar year and a prior mental disorder.
2. End of calendar year is 31 August 2021 for the year 2021

**eFigure 3:** **Age-standardized Annual incidence^a^ rate (Per 100,000 Population) among people with opioid use disorder (first indication) and concurrent mental disorders (stratified by sex) and 95% Wald Confidence interval (CI)**  **in British Columbia, 2013-2021^b^**

**
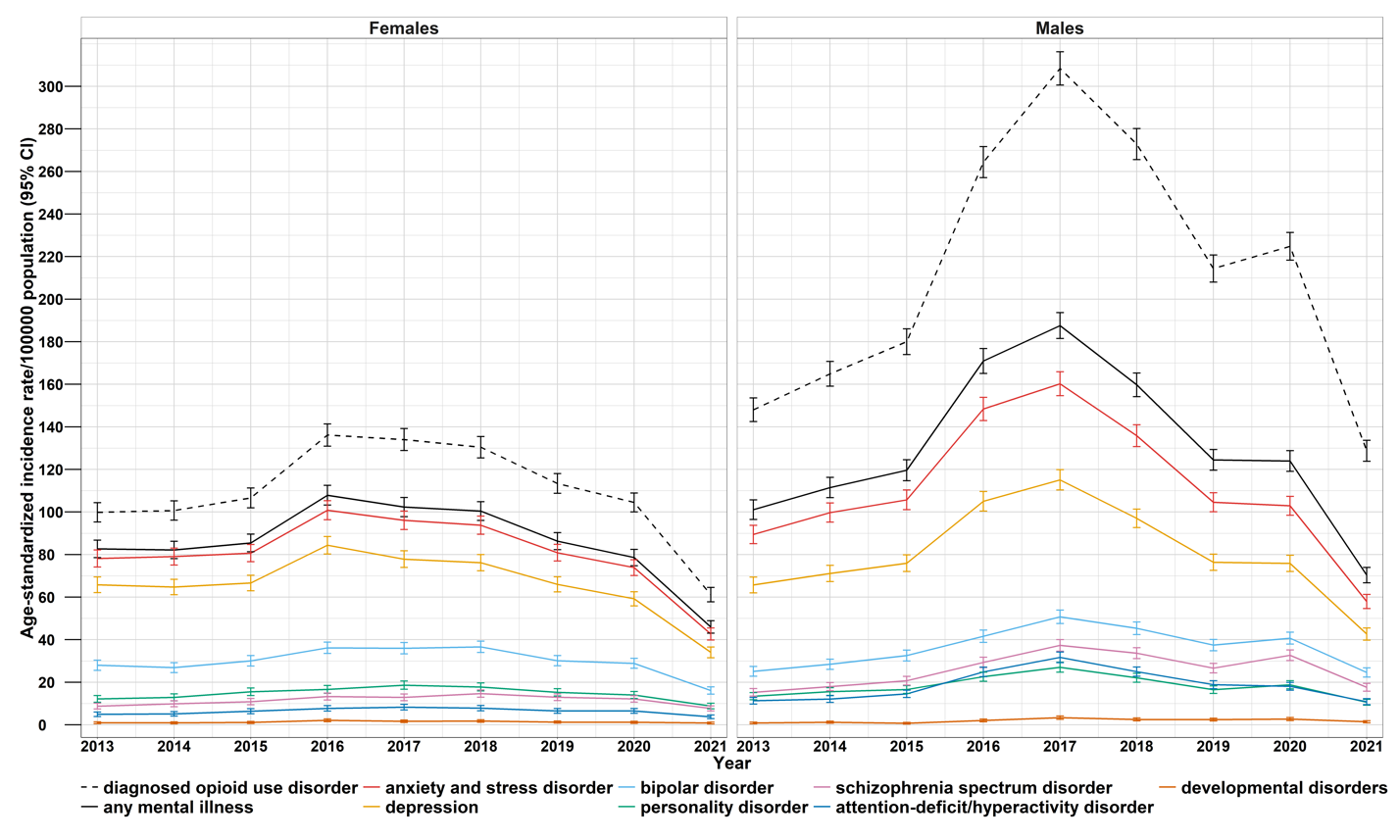
**

1. People who were in the follow up within the calendar year and diagnosed with opioid use disorder for the first time within the calendar year and prior mental disorder.

b. End of calendar year is 31 August 2021 for the year 2021.

**References**

1. Nosyk B, Colley G, Yip B, Chan K, Heath K, Lima VD, et al. Application and validation of case-finding algorithms for identifying individuals with human immunodeficiency virus from administrative data in British Columbia, Canada. PloS one. 2013;8(1):e54416.

2. Shah H, Bilodeau M, Burak KW, Cooper C, Klein M, Ramji A, et al. The management of chronic hepatitis C: 2018 guideline update from the Canadian Association for the Study of the Liver. CMAJ. 2018;190(22):E677-E87.

3. Perrone V, Sangiorgi D, Buda S, Degli Esposti L. Disease progression and health care resource consumption in patients affected by hepatitis C virus in real practice setting. Clinicoecon Outcomes Res. 2016;8:591-7.

4. Kim D, Li AA, Gadiparthi C, Khan MA, Cholankeril G, Glenn JS, et al. Changing Trends in Etiology-Based Annual Mortality From Chronic Liver Disease, From 2007 Through 2016. Gastroenterology. 2018;155(4):1154-63.e3.

5. Degenhardt L, Randall D, Hall W, Law M, Butler T, Burns L. Mortality among clients of a state-wide opioid pharmacotherapy program over 20 years: risk factors and lives saved. Drug Alcohol Depend. 2009;105(1-2):9−15.

6. Centers for Disease Control and Prevention. Alcohol-Related ICD Codes 2020 [Available from: <https://www.cdc.gov/alcohol/ardi/alcohol-related-icd-codes.html>.

7. Quan H, Sundararajan V, Halfon P, Fong A, Burnand B, Luthi JC, et al. Coding algorithms for defining comorbidities in ICD-9-CM and ICD-10 administrative data. Medical care. 2005;43(11):1130–9.

8. Tian TY, Zlateva I, Anderson DR. Using electronic health records data to identify patients with chronic pain in a primary care setting. J Am Med Inform Assoc. 2013;20(e2):e275-80.
